# Risk factors for thrombotic events in Korean patients with systemic lupus erythematosus

**DOI:** 10.1038/s41598-021-03074-5

**Published:** 2021-12-07

**Authors:** Dong-Jin Park, Chang-Seok Yoon, Sung-Eun Choi, Haimuzi Xu, Ji-Hyoun Kang, Shin-Seok Lee

**Affiliations:** Division of Rheumatology, Department of Internal Medicine, Chonnam National University Medical School & Hospital, 42 Jebong-ro, Dong-gu, Gwangju, 61469 Republic of Korea

**Keywords:** Rheumatology, Risk factors

## Abstract

Thrombotic events (TE), including deep vein thrombosis, stroke, and myocardial infarction, occur in 30–40% of patients with systemic lupus erythematosus (SLE) resulting in substantial morbidity and mortality. We explored the risk factors for TE in SLE patients. We analyzed data obtained during a prospective cohort based on the KORean lupus NETwork (KORNET) registry, and enrolled 259 SLE patients with clinical data available at the onset of SLE. TE was defined as the presence of arterial or venous thrombosis. Multivariate Cox-proportional hazards analysis was performed to investigate risk factors for TE. During a mean follow-up of 103.3 months (SD 53.4), 27 patients (10.4%) had a TE. In multivariate analysis, hypertension (hazard ratio [HR] 7.805, 95% confidence interval [CI]: 1.929–31.581; *P* = 0.004), anti-phospholipid syndrome (APS) (HR 12.600, 95% CI: 4.305–36.292; *P* < 0.001), mean daily prednisolone > 5 mg/day (HR 3.666, 95% CI: 1.318–10.197; *P* = 0.013), and SLICC/ACR Damage Index (SDI) score (HR 1.992, 95% CI: 1.465–2.709; *P* < 0.001) were significantly associated with the development of TE in SLE patients. Instead, use of an ACEi or ARB (HR 0.159, 95% CI: 0.043–0.594; *P* = 0.006) was a protective factor against TE development in these patients. In conclusion, hypertension, higher mean daily dose of prednisolone, diagnosis of APS, and higher SDI were risk factors for TE in patients with SLE. On the other hand, the use of an ACEi or ARB was associated with a reduced risk of TE.

## Introduction

Systemic lupus erythematosus (SLE) is a chronic autoimmune disease characterized by a variety of clinical manifestations, the production of various autoantibodies and immune complexes, and multiple organ involvement including glomerulonephritis, neuropsychiatric and hematological manifestations^1^. Although long-term prognosis and survival in patients with SLE have improved over the last few decades, mortality still remains higher than in the general population. In a multicenter cohort study of 1,000 patients from seven European countries, the most common causes of death were SLE disease activity, thrombotic events (TE), and infection^2,3^. The proportion of early deaths caused by active SLE or infection has decreased over time, probably reflecting the evolution of the diagnosis and treatment of SLE^3^. Accordingly, TE, such as myocardial infarction and stroke, are becoming the leading causes of both morbidity and mortality in SLE^2^.

TE occur at a higher rate in patients with SLE than in the general population (10–30 vs. 1–5 cases per 1,000 person-years, respectively)^2,4–6^. The presence of anti-phospholipid antibodies (aPL) is a well-known risk factor for TE in SLE^7^. In addition, a recent study showed that the pattern of aPL positivity is also associated with thrombosis in SLE patients^8^. However, other causative factors of TE in patients with SLE have also been investigated. Age, poverty, smoking, accumulated damage, and higher doses of glucocorticoids were shown to be independently associated with a shorter time to the first TE in the LUMINA (Lupus in Minority: NAture vs. Nurture) cohort^9^. In a study population comprised of patients of three different ethnicities (Chinese, African American, and white), ethnicity, male sex, lower levels of high-density lipoprotein (HDL) cholesterol, oral ulcers, serositis, renal disease, and hemolytic anemia were shown to be associated with TE^5^. Sarabi et al*.* reported that older age at SLE diagnosis, shorter disease duration, disease activity, smoking, and accumulated damage were associated with TE^10^. However, the interpretability of previous studies is limited by their relatively small sample sizes and variation in study populations and/or the laboratory assays used. Furthermore, few studies have assessed the risk factors for TE in Asian SLE patients. As TE is associated with poor outcomes and impaired quality of life in SLE patients, identifying risk factors for TE will help clinicians make informed decisions regarding the management of patients at risk of thrombosis. This study was performed to identify risk factors for TE in Korean patients with SLE, to inform strategies for prevention and treatment.

## Methods

### Population and study design

This study forms part of a prospective longitudinal study based on the KORean lupus NETwork (KORNET) registry, and involved retrospective analysis of those data. KORNET is a nationwide, multicenter, hospital-based registry used to assess clinical manifestations and long-term outcomes in Korean patients with SLE^11^. To evaluate the prevalence and risk factors for TE, we enrolled SLE patients from among those registered with KORNET who visited Chonnam National University Hospital (CNUH) within 6 months of diagnosis. All patients fulfilled the 1997 revised criteria for the classification of SLE^12^.

All patients were followed up at 3–6-month intervals, from the time of diagnosis for at least 1 year, via the KORNET database. Patients with inadequate medical records or a follow-up period < 1 year were excluded from this study. Finally, 259 unselected consecutive and ethnically homogeneous Korean patients with SLE were enrolled in the study. The electronic case report form (CRF) was developed, and the KORNET data managed, using the Internet-based Clinical Research and Trial (iCReaT) data management system (http://icreat.nih.go.kr) established by the Centers for Disease Control and Prevention, Ministry of Health and Welfare, Republic of Korea (iCReaT Study No. C140018). The study received approval from the Institutional Review Board of Chonnam National University Hospital (CNUN-2014–239) and all patients provided informed consent at the time of registry enrolment. This study was conducted in accordance with the Declaration of Helsinki and the Good Clinical Practice guidelines.

### Patient data collection

Baseline characteristics were collected at the time of diagnosis of SLE. Demographic data included age at onset of SLE, sex, duration of SLE, smoking history, and comorbidities at the time of SLE diagnosis. Hypertension was defined as systolic blood pressure ≥ 140 mm Hg and/or diastolic blood pressure ≥ 90 mm Hg on two or more occasions, and/or patient self-reported use of antihypertensive medications. Diabetes mellitus was defined as a history of fasting glucose level ≥ 140 mg/dL or the use of insulin or hypoglycemic medications.

Clinical manifestations of SLE, including malar rash, discoid rash, photosensitivity, oral ulcers, arthritis, serositis (pleuritis and pericarditis), renal disorder, central nervous system (CNS) involvement, and hematological disorders, were defined according to the 1997 Revised Criteria for Classification of SLE^12^. Other signs and symptoms related to SLE were defined as follows: proteinuria > 0.5 g/day or > 3 + if quantification was not performed, hematuria [≥ 5 red blood cells/high-power field, after ruling out other possible causes] and pyuria [≥ 5 white blood cells (WBCs)/high-power field, after ruling out infection]. Concomitant diseases were also investigated. Raynaud’s phenomenon was diagnosed based on discoloration of the fingers and/or toes induced by exposure to cold and/or an emotional event, with a clear two-phase color change. Anti-phospholipid syndrome (APS) was defined according to the revised APS classification criteria^13^, and associated Sjögren’s syndrome was diagnosed based on the 2012 American College of Rheumatology (ACR) classification criteria^14^. Interstitial lung disease was diagnosed based on radiological findings, such as chest X-ray and chest computed tomography (CT). A history of thyroid disease, such as hyperthyroidism or hypothyroidism, was also determined based on a review of the medical records. Disease activity was evaluated using the SLE disease activity index (SLEDAI)-2K^15^, and organ damage was assessed based on the Systemic Lupus International Collaborating Clinics (SLICC)//ACR Damage Index (SDI)^16^ by a trained rheumatologist. SDI was measured before development of TE, or at the last visit.

We obtained laboratory findings, such as WBC count, hemoglobin concentration, platelet level, serum albumin level, erythrocyte sedimentation rate (ESR), C-reactive protein (CRP) level, total cholesterol level, serum creatinine level, urinalysis, and level of proteinuria (g/day) at the time of SLE diagnosis. Serological markers, such as anti-nuclear antibody (ANA), complement (C3 and C4), and various autoantibodies, including anti-dsDNA, Smith (Sm), ribonucleoprotein (RNP), Ro, and La, were assessed by enzyme-linked immunosorbent assays (ELISA). We also assessed the presence of lupus anticoagulant (LAC), anti-cardiolipin (aCL), and anti-beta2-glycoprotein I (β2GPI). LAC was measured using the modified Russell’s viper venom time (RVVT) test, with confirmatory mixing studies. IgG/M aCL and IgG/M anti-β2GPI antibodies were analyzed by ELISA, and the results were considered positive if the titer was medium to high. aPL positivity was confirmed after 12 weeks, if the patient had at least one of these autoantibodies.

We investigated the medications taken for more than 6 months to treat SLE, such as hydroxychloroquine (HCQ), prednisolone (> 5 mg/day), and immunosuppressive agents. We also determined whether HCQ was taken continuously (defined as > 80% of the follow-up period) after diagnosis of SLE. Use of an angiotensin-converting enzyme inhibitor (ACEi) or angiotensin receptor blocker (ARB), and anti-coagulation or anti-platelet medication, was also reviewed.

### Outcome variables

The main outcome of this study was the development of TE (defined as clinical signs and symptoms of vascular occlusion confirmed by clinical studies) after diagnosis of SLE. The TE included ischemic heart disease (including myocardial infarction and/or unstable/stable angina pectoris based on clinical diagnosis and/or ischemic changes in the electrocardiogram and/or specific changes in cardiac enzymes and/or typical findings in a coronary angiography), cerebral vascular accident (based on a unequivocal previous diagnosis or on the presence of clinical manifestations and/or supported by an imaging procedure (i.e., CT angiography or magnetic resonance angiography [MRA]), deep vein thrombosis (DVT), and peripheral artery disease evaluated with Doppler ultrasound, CT, or angiography.

### Statistical analysis

Statistical analyses were performed using SPSS software (ver. 21.0; SPSS Inc., Chicago, IL, USA). Values are expressed as means ± standard deviation (SD) for continuous variables and percentages for categorical variables. The patients were divided into two groups: patients with TE (TE group) and patients without TE (non-TE group). Continuous variables were compared with the nonparametric Mann–Whitney U test and categorical variables were compared using the Chi-square test. Cox proportional hazard regression analysis was performed to identify predictors of TE after diagnosis of SLE. We also performed multivariate analysis, including age, gender, disease duration and variables with a *P*-value < 0.10 on the nonparametric Mann–Whitney U test or Chi-square test. There were missing data on cholesterol profiles at the time of enrolment in the KORNET registry. Therefore, multiple imputations were performed for this variable in these cases. In all analyses, *P* < 0.05 was taken to indicate statistical significance.

## Results

A total of 259 patients with SLE were enrolled in this study. The mean age of the patients was 34.0 years (SD: 13.7 years), and 239 (92.3%) were female. Of these patients, 27 (10.4%) suffered TE during the follow-up of 103.3 ± 53.4 months. As shown in Table 1, the most common TE after diagnosis of SLE was stroke (15 cases), followed by venous thrombosis (4 cases), myocardial infarction (4 cases), angina (3 cases), and peripheral arterial thrombosis (1 case). Three patients died as a result of TE; two due to myocardial infarction and one from stroke.

**Table 1:** Thrombotic events during follow-up in patients with systemic lupus erythematosus.

	Thrombotic events (*N* = 27)
**Total thrombotic cases**	27
Stroke	15
Venous thrombosis	4
Myocardial infarction	4
Angina	3
Peripheral arterial thrombosis	1
Death due to thrombotic events^a^	3

^a^Two cases died due to myocardial infarction and one case due to stroke.

The baseline demographic and clinical characteristics of the patients are shown in Table 2. Patients in the TE group were more likely to have hypertension at the time of enrollment than those in the non-TE group (63.0% and 34.9%, respectively; *P* = 0.004). However, there were no significant differences in sex ratio, symptom duration, smoking history, presence of diabetes mellitus, or SLEDAI score at the time of SLE onset between the two groups. With regard to clinical features, neuropsychiatric lupus (18.5% and 6.9%, respectively; *P* = 0.036) and proteinuria (55.6% and 34.9%, respectively; *P* = 0.036) were more prevalent in the TE than non-TE group. On the other hand, the TE group showed a trend toward a lower rate of oral ulcers (*P* = 0.096), although this was not significantly associated with the development of TE in either group. With regard to concomitant diseases, APS was observed more frequently in the TE than non-TE group (22.2% and 3.0%, respectively; *P* = 0.001).

**Table 2:** Baseline demographic and clinical characteristics of SLE patients.

	All (*N* = 259)	Thrombotic events (*n* = 27)	No event (*n* = 232)	*P*-value
Age at onset of SLE, years	34.0 ± 13.7	35.1 ± 14.2	33.9 ± 13.6	0.646
Females (%)	239 (92.3)	24 (88.9)	215 (92.7)	0.486
Disease duration, months	2.87 ± 5.44	3.68 ± 4.79	2.77 ± 5.51	0.412
Ever smoker (former or current) (%)	15 (5.8)	3 (11.1)	12 (5.2)	0.196
Diabetes mellitus (%)	6 (2.3)	0 (2.7)	6 (2.6)	0.513
Hypertension (%)	98 (37.8)	17 (63.0)	81 (34.9)	0.004
SLEDAI-2K score	10.7 ± 6.18	12.1 ± 6.53	10.5 ± 6.13	0.219
**Met the ACR-97 criteria (%)**				
Malar rash	131 (60.6)	10 (37.0)	121 (52.2)	0.137
Discoid rash	26 (10.0)	1 (3.7)	25 (10.8)	0.214
Photosensitivity	81 (31.3)	7 (25.9)	74 (31.9)	0.527
Oral ulcer	47 (18.1)	2 (7.4)	45 (19.4)	0.096
Arthritis	114 (44.0)	10 (37.0)	104 (44.8)	0.440
Pleuritis	31 (12.0)	2 (7.4)	29 (12.5)	0.344
Pericarditis	23 (8.9)	4 (14.8)	19 (8.2)	0.206
CNS involvement	21 (8.1)	5 (18.5)	16 (6.9)	0.036
Renal involvement				
Proteinuria	96 (37.1)	15 (55.6)	81 (34.9)	0.036
Hematuria	70 (32.0)	13 (48.1)	70 (30.2)	0.058
Pyuria	67 (25.9)	9 (33.3)	58 (25.0)	0.349
Hematological involvement				
Lymphopenia	201 (77.6)	20 (74.1)	181 (78.0)	0.642
Anemia	78 (30.1)	9 (33.3)	69 (29.7)	0.700
Thrombocytopenia	66 (25.5)	8 (29.6)	58 (25.0)	0.601
**Concomitant disease (%)**				
Interstitial lung disease	10 (3.9)	2 (7.4)	8 (3.4)	0.280
Raynaud’s phenomenon	60 (23.2)	4 (14.8)	56 (24.1)	0.202
Anti-phospholipid syndrome	13 (5.0)	6 (22.2)	7 (3.0)	0.001
Associated Sjögren’s syndrome	27 (10.4)	4 (14.8)	23 (9.9)	0.305
Thyroid disease	20 (7.7)	1 (3.7)	19 (8.2)	0.357

Except where otherwise indicated, data are presented as the mean ± standard deviation.

Abbreviations: CNS, central nervous system; SLE, systemic lupus erythematosus; SLEDAI, systemic lupus erythematosus disease activity index.

The laboratory findings of patients with and without TE are compared in Table 3. Patients in the TE group had higher serum total cholesterol levels than those in the non-TE group (194.2 ± 64.4 and 167.9 ± 53.2, respectively; *P* = 0.046). Furthermore, patients in the TE group showed a trend toward a higher serum creatinine level than those in the non-TE group (*P* = 0.074), although this was not significantly associated with TE. Comparison of the baseline serological tests of the two groups indicated that only aPL positivity was significantly associated with TE (37.0% and 19.2% in the TE and non-TE group, respectively; *P* = 0.032). Other laboratory findings, such as the WBC count, hemoglobin and platelet levels, autoantibodies other than aPL, and complement level were not different between the two groups.

**Table 3:** Comparison of laboratory findings between SLE patients with and without thrombotic events.

	All (*N* = 259)	Thrombotic events (*n* = 27)	No event (*n* = 232)	*P*-value
**Laboratory findings**				
White blood cells, 1,000/mm^3^	4.88 ± 2.66	5.37 ± 2.43	4.82 ± 2.68	0.148
Lymphocytes, mm^3^	1.16 ± 0.66	1.26 ± 0.62	1.15 ± 0.64	0.373
Hemoglobin, g/dL	11.3 ± 6.67	11.1 ± 2.59	11.4 ± 6.98	0.459
Platelet, × 10^3^/μL	183.1 ± 96.7	179.7 ± 108.2	183.5 ± 95.5	0.885
ESR, mm/h	48.5 ± 34.1	40.0 ± 29.9	49.7 ± 34.5	0.200
CRP, mg/dL	1.89 ± 2.45	0.09 ± 1.47	1.22 ± 2.54	0.241
Serum creatinine, mg/dL	0.78 ± 0.85	1.00 ± 1.05	0.75 ± 0.82	0.074
Total cholesterol, mg/dL	170.7 ± 54.9 (*n* = 249)	194.2 ± 64.4 (*n* = 27)	167.9 ± 53.2 (*n* = 222)	0.046
LDL-cholesterol, mg/dL	97.7 ± 36.7 (*n* = 187)	105.3 ± 36.8 (*n* = 20)	96.7 ± 36.7 (*n* = 167)	0.335
**Autoantibodies (%)**				
Anti-nuclear	227 (91.2)	23 (88.5)	204 (91.5)	0.608
Anti-Sm	78 (30.2)	7 (25.9)	71 (30.7)	0.607
Anti-dsDNA	147 (56.8)	13 (48.1)	134 (57.8)	0.340
Anti-RNP	109 (42.2)	9 (33.3)	100 (43.3)	0.322
Anti-Ro/SS-A	160 (61.8)	18 (66.7)	142 (61.2)	0.581
Anti-La/SS-B	73 (28.2)	9 (33.3)	64 (27.6)	0.530
aPL	54 (21.1)	10 (37.0)	44 (19.2)	0.032
**Complement level (%)**				
Decreased C3 level	178 (68.1)	16 (59.3)	162 (69.8)	0.262
Decreased C4 level	124 (47.9)	11 (40.7)	113 (48.7)	0.282

Except where otherwise indicated, data are presented as the mean ± standard deviation.

aPL, anti-phospholipid antibody; ESR, erythrocyte sedimentation rate; CRP, C-reactive protein; SLE, systemic lupus erythematosus.

Table 4 shows the treatment-related factors, including medication taken before the onset of TE and changes in SDI. With regard to medications, the TE group had a higher proportion of patients with a mean daily prednisolone dose > 5 mg/day than the non-TE group (76.9% and 31.6%, respectively; *P* < 0.001). In addition, the rate of anticoagulation or antiplatelet drug use was higher in the group than non-TE group (37.0% and 19.4%, respectively; *P* = 0.034). Although not significant (*P* = 0.068), the rate of use of an ACEi or ARB tended to be higher in the TE than non-TE group. HCQ and immunosuppressive agents, including intravenous cyclophosphamide, taken during follow-up were not associated with the occurrence of TE in patients with SLE. Organ damage other than thrombotic complications at the last visit, as measured by the SDI, was higher in the TE than non-TE group (2.07 ± 1.43 and 0.86 ± 0.98, respectively; *P* < 0.001).

**Table 4:** Comparison of treatments and SDI scores between SLE patients with and without thrombotic events.

	All (*n* = 259)	Thrombotic events (*n* = 27)	No event (*n* = 232)	*P*-value
**Medication (%)**				
Mean daily prednisolone > 5 mg/day	93 (36.2)	20 (76.9)	73 (31.6)	< 0.001
Hydroxychloroquine > 80% during follow-up	224 (86.5)	22 (81.5)	202 (87.1)	0.422
Immunosuppressive agents	145 (56.0)	19 (70.4)	126 (54.3)	0.112
Cyclophosphamide	55 (21.2)	7 (25.9)	48 (20.7)	0.340
ACEi or ARB	93 (34.1)	14 (51.9)	79 (34.1)	0.068
Statin	41 (15.8)	6 (22.2)	35 (15.1)	0.336
Anticoagulation or antiplatelet drugs	55 (21.2)	10 (37.0)	45 (19.4)	0.034
SDI^a^	0.99 ± 1.10	2.07 ± 1.43	0.86 ± 0.98	< 0.001

^a^SDI was measured before development of thrombotic events or at the last visit.

Abbreviations: ACEi, angiotensin-converting enzyme inhibitor; ARB, angiotensin receptor blocker; SDI, Systemic Lupus International Collaborating Clinics/American College of Rheumatology (SLICC/ACR) Damage Index; SLE, systemic lupus erythematosus.

Univariate and multivariate Cox proportional hazards regression analyses were performed to identify predictors of TE in patients with SLE (Table 5). We included variables with a *P*-value < 0.10 in the Mann–Whitney U and Chi-square tests. Based on univariate analysis, several factors such as hypertension, CNS involvement, proteinuria, aPL antibody, total cholesterol, APS, mean daily prednisolone > 5 mg/day, use of anti-coagulation or anti-platelet drugs, use of an ACEi or ARB, and the SDI at the last visit were associated with the development of TE in patients with SLE. In multivariate analysis, hypertension (hazard ratio [HR] = 7.805, 95% confidence interval [CI]: 1.929–31.581; *P* = 0.004), APS (HR = 12.600, 95% CI: 4.305–36.292; *P* < 0.001), mean daily prednisolone > 5 mg/day (HR = 3.666, 95% CI: 1.318–10.197; *P* = 0.013), and SDI (not including thrombotic complications) at the last visit (HR = 1.992, 95% CI: 1.465–2.709; *P* < 0.001) predicted TE in patients with SLE. On the other hand, use of an ACEi or ARB (HR = 0.159, 95% CI: 0.043–0.594; *P* = 0.006) was a protective factor against TE development in patients with SLE.

**Table 5:** Uni- and multivariate Cox-proportional hazard regression analyses of predictors of the development of thrombotic events in patients with SLE.

Variables	Univariate analysis		Model 1^a^		Model 2^b^	
	HR	*P*-value	HR	*P*-value	HR	*P*-value
Hypertension	3.938 (1.527–10.154)	0.005	2.857 (1.247–6.547)	0.013	7.805 (1.929–31.581)	0.004
Anti-phospholipid syndrome	9.828 (3.798–25.434)	< 0.001	7.291 (2.868–18.536)	< 0.001	12.600 (4.305–36.292)	< 0.001
Mean daily prednisolone > 5 mg/day	14.255 (5.161–39.377)	< 0.001	9.039 (3.455–23.646)	< 0.001	3.666 (1.318–10.197)	0.013
ACEi or ARB	2.344 (0.988–5.565)	0.053	2.002 (0.925–4.334)	0.078	0.159 (0.043–0.594)	0.006
SDI^c^	1.997 (1.463–2.725)	< 0.001	1.980 (1.512–2.592)	< 0.001	1.992 (1.465–2.709)	< 0.001

ACEi, angiotensin-converting enzyme inhibitor; aPL, anti-phospholipid antibody; ARB, angiotensin receptor blocker; HR, hazard ratio; SDI, Systemic Lupus International Collaborating Clinics/American College of Rheumatology (SLICC/ACR) Damage Index; SLE, systemic lupus erythematosus.

^a^Multivariate Cox-proportional hazard regression analysis adjusted for age, gender and disease duration.

^b^Multivariate Cox-proportional hazard regression analysis adjusted for age, gender, and disease duration, and variables significant at *P* < 0.10 in univariate analyses, including hypertension, central nervous system involvement, proteinuria, anti-phospholipid antibody, total cholesterol, anti-phospholipid syndrome, mean daily prednisolone > 5 mg/day, anticoagulation or antiplatelet drugs, ACEi or ARB and SDI.

^c^SDI was measured before the occurrence of thrombotic events or at the last visit.

We conducted further analyses including SLE patients without aPL or previous APS. In the multivariable Cox proportional hazard model, hypertension (HR = 19.037, 95% CI: 3.530–102.67; *P* = 0.001), mean daily prednisolone > 5 mg/day (HR = 4.056, 95% CI: 1.029–15.992; *P* = 0.045), SDI (not including thrombotic complications) at the last visit (HR = 2.024, 95% CI: 1.364–3.002; *P* < 0.001), and use of an ACEi or ARB (HR = 0.087, 95% CI: 0.020–0.374; *P* = 0.001) remained as independent predictors of TE development in patients with SLE (data not shown).

## Discussion

In the present study, 27 of 259 (10.4%) patients with SLE developed various TE during the follow-up period of 103.3 ± 53.4 months. We found that hypertension, previous APS, higher daily mean dose of prednisolone, and SDI predicted TE in patients with SLE. On the other hand, use of an ACEi or ARB protected against TE in patients with SLE.

While there have been significant improvements in the treatment of SLE and its associated comorbidities, the development of TE during the course of the disease remains a major concern in the management of patients with SLE^7^. In fact, thrombosis is one of the most frequent complications seen in patients with SLE, and significantly affects morbidity and mortality^17,18^. However, the incidence and risk factors of thrombosis in SLE patients have not been fully elucidated. In fact, ethnic differences have been reported in the incidence of TE in patients with SLE, suggesting that Asian ethnicity may be an independent predictor of these events^5^.

The prevalence of TE was relatively high in the present study, even though the mean age of our cohort at the time of enrollment was 34.0 years. Thrombotic complications have been reported at a higher frequency (range: 10–35%), and at a younger age, in SLE patients than the general population^5,9,19,20^. In fact, Manzi et al*.* showed that young premenopausal women with SLE in the 25–44 years age group had a significantly higher incidence of cardiovascular thrombosis than the general population^21^. In addition, Ward et al*.* reported that young women with SLE aged 18–44 years are hospitalized with myocardial infarction or a cerebrovascular event almost twice as often as the general population^22^. Therefore, identifying SLE patients at higher risk of TE, even those younger than 40 years of age, is essential to limit the irreversible damage and preserve quality of life.

In our cohort, the prevalence of hypertension was high (37.8%) and it was significantly associated with TE in SLE patients. Hypertension is recognized as an important contributor to not only organ damage accrual^23^ but also thrombotic events, such as heart attacks, stroke, and DVT in SLE patients^24^. Numerous studies have shown that effective control of hypertension reduces these complications, by 20–25% for myocardial infarction and 30–40% for stroke^24,25^. The incidence of hypertension is higher in SLE patients than healthy controls, especially in female SLE patients younger than 40 years (40% and 11%, respectively; *P* < 0.001)^26^. Our study showed that blood pressure monitoring in conjunction with effective blood pressure control (including via medication, education, and lifestyle counseling) is essential for SLE patients at increased risk of TE.

In the present study, despite treatment with anticoagulants and/or antiplatelet drugs, a previous diagnosis of APS was significantly associated with TE development. Even without considering APS, SLE is well known to be an independent risk factor for the development of TE, due to the potential for lupus-associated endothelial activation and damage^27^. Furthermore, cardiovascular problems such as hypertension and hyperlipemia, which occur during the course of SLE, may increase the risk of thrombosis^28,29^. Against this background, the frequency of thrombosis is higher in APS associated with SLE than primary APS^28^. In fact, Zaldívar-Alcántara et al. reported that a previous diagnosis of APS was the most important risk factor for thrombotic complications in patients with SLE^30^. Our study also showed that APS is a major risk factor for TE during the course of SLE. Therefore, clinicians should be aware of the need for aggressive monitoring of thrombosis and secondary prophylactic therapy in SLE patients with previous APS.

In the present study, a higher mean daily glucocorticoid dose was a clinical predictor of TE in patients with SLE. Previous studies have shown that both the cumulative dose and higher daily dose are associated with thrombosis in patients with SLE^9,31^. The increased risk of thrombosis related to glucocorticoid use is probably mediated by endothelial damage, accelerated atherosclerosis and abnormalities in the coagulation cascade^31^. Glucocorticoid use could accelerate thrombocytosis and hyperlipidemia^32–34^. Therefore, avoidance of prolonged high doses of corticosteroids in SLE patients is important to minimize the risk of thrombotic complications.

We also showed that the accumulation of organ damage during the disease course was a strong clinical predictor of TE. Roman et al. reported that more severe organ damage was associated with an increased risk of thrombotic complications, such as cardiovascular disease, in patients with SLE^35^. Similarly, in the LUMINA cohort, higher SDI was independently associated with a shorter time to the first TE^9^. Both higher mean daily glucocorticoid dose and an accumulation of organ damage are plausible proxies for SLE activity. In fact, the glucocorticoid dose is usually determined according to SLE activity during the course of the disease^36^. In addition, disease activity during the course of SLE, as measured by the mean total British Isles Lupus Assessment Group Index (BILAG), was associated with a risk of subsequent organ damage^37^. Our study suggested that aggressive monitoring and judicious use of immunosuppressive agents for prompt control of disease activity are important to minimize the risk of thrombosis in patients with SLE.

Interestingly, we found that use of an ACEi or ARB reduced the risk of thrombosis in patients with SLE. Current data suggest that renin–angiotensin–aldosterone system (RAAS) blockers can reduce the risk of thrombosis at least partly independently of blood pressure control. In fact, there is accumulating evidence suggesting a prothrombotic effect of RAAS, reflected in vascular inflammation and platelet activation^38^. In this context, ACEi and ARBs, which inhibit the RAAS, exert antithrombotic effects by decreasing platelet activation and improving endothelial function^39^. In fact, the use of an ACEi or ARB may reduce the risk of TE in patients with atherosclerotic disease^40^. Moreover, recent evidence suggested that modulation of RAAS along with blood pressure reduction is probably the optimal means of reducing the risk of cardiovascular thrombosis^24,41^. However, there is little evidence of antithrombotic effects of ACEi or ARBs in patients with SLE. To date, only one study has shown that the use of an ACEi was associated with a reduced risk of developing thrombotic complications (odds ratio = 0.19; *P* = 0.04) in patients with SLE^30^. In conclusion, although this possible antithrombotic effect of ACEi or ARB use in patients with SLE should be confirmed in further prospective studies, our findings indicated that an ACEi or ARB should be considered as the first-line antihypertensive medication to prevent thrombosis in patients with SLE at high risk of TE.

This study had several limitations. First, it was a single hospital-based observational study, and the number of patients enrolled was relatively small so the results may not be generalizable. In addition, in our SLE cohort, traditional risk factors other than hypertension, such as age, sex, smoking history, and diabetes mellitus, were not associated with the development of TE. As thrombosis shows very slow progression, the relatively short-term follow-up of our study (< 10 years) may not have been adequate to assess the effects of these factors on the development of TE. Furthermore, our sample size may not have been sufficient to demonstrate the contributions of these risk factors to thrombosis. In fact, in SLE patients, the risk of thrombosis cannot be fully explained by baseline values of the traditional risk factors^42^. Similarly, with the exception of ACEi and ARBs, no medications (such as antiplatelet drugs) showed protective effects in our patients. Antiplatelet drugs, including aspirin, were associated with a reduced risk of thrombotic complications in SLE patients^43,44^. However, in this study, this protective effect could have been substantially offset by other factors. Finally, this open, observational study had inherent limitations, such as a lack of exposure randomization, lack of a control group, and the possibility of selection bias and confounding factors. Therefore, although we believe that our study design does not weaken the value of our findings, future research should include a control group or perform randomized controlled trials to confirm our results.

## Conclusion

In summary, we investigated the risk factors for TE in patients with SLE. This study showed that TE develops in the presence of multiple risk factors during the disease course of SLE. These findings suggest that multifactorial treatment approaches are required for SLE patients to reduce the likelihood of TE, which are potentially devastating complications of SLE.

## Data Availability

Full original protocol and dataset can be accessed upon request for academic researchers by contacting Professor Shin-Seok Lee (shinseok@chonnam.ac.kr).
